# Integrating Early Growth Information to Monitor Winter Wheat Powdery Mildew Using Multi-Temporal Landsat-8 Imagery

**DOI:** 10.3390/s18103290

**Published:** 2018-09-30

**Authors:** Huiqin Ma, Yuanshu Jing, Wenjiang Huang, Yue Shi, Yingying Dong, Jingcheng Zhang, Linyi Liu

**Affiliations:** 1 Collaborative Innovation Center on Forecast and Evaluation of Meteorological Disasters, Nanjing University of Information Science & Technology, Nanjing 210044, China; mahq0712@nuist.edu.cn; 2 Key Laboratory of Digital Earth Science, Institute of Remote Sensing and Digital Earth, Chinese Academy of Sciences, Beijing 100094, China; shiyue@radi.ac.cn (Y.S.); dongyy@radi.ac.cn (Y.D.); liuly35@radi.ac.cn (L.L.); 3 Aerospace Information Research Institute, Chinese Academy of Sciences, Beijing 100094, China; 4 University of Chinese Academy of Sciences, Beijing 100049, China; 5 College of Life Information Science and Instrument Engineering, Hangzhou Dianzi University, Hangzhou 310018, China; zhangjc_rs@163.com

**Keywords:** winter wheat, powdery mildew, monitoring, multi-temporal, remote sensing

## Abstract

Powdery mildew is one of the dominant diseases in winter wheat. The accurate monitoring of powdery mildew is important for crop management and production. Satellite-based remote sensing monitoring has been proven as an efficient tool for regional disease detection and monitoring. However, the information provided by single-date satellite scene is hard to achieve acceptable accuracy for powdery mildew disease, and incorporation of early period contextual information of winter wheat can improve this situation. In this study, a multi-temporal satellite data based powdery mildew detecting approach had been developed for regional disease mapping. Firstly, the Lansat-8 scenes that covered six winter wheat growth periods (expressed in chronological order as periods 1 to 6) were collected to calculate typical vegetation indices (VIs), which include disease water stress index (DSWI), optimized soil adjusted vegetation index (OSAVI), shortwave infrared water stress index (SIWSI), and triangular vegetation index (TVI). A multi-temporal VIs-based *k*-nearest neighbors (KNN) approach was then developed to produce the regional disease distribution. Meanwhile, a backward stepwise elimination method was used to confirm the optimal multi-temporal combination for KNN monitoring model. A classification and regression tree (CART) and back propagation neural networks (BPNN) approaches were used for comparison and validation of initial results. VIs of all periods except 1 and 3 provided the best multi-temporal data set for winter wheat powdery mildew monitoring. Compared with the traditional single-date (period 6) image, the multi-temporal images based KNN approach provided more disease information during the disease development, and had an accuracy of 84.6%. Meanwhile, the accuracy of the proposed approach had 11.5% and 3.8% higher than the multi-temporal images-based CART and BPNN models’, respectively. These results suggest that the use of satellite images for early critical disease infection periods is essential for improving the accuracy of monitoring models. Additionally, satellite imagery also assists in monitoring powdery mildew in late wheat growth periods.

## 1. Introduction

Powdery mildew (*Blumeria graminis*) is one of the most destructive foliar diseases infecting winter wheat and occurs in areas with cool or maritime climates [1]. The disease interferes with the plant’s normal source-sink relationships. It also changes the translocation and distribution of photoassimilate, causing changes in grain starch and protein composition [2]. This in turn results in a reduction in wheat quality and yield [3]. According to the statistics by China’s National Agricultural Technology Extension and Service Center (NATESC), the annual average outbreak area for powdery mildew was 10 million ha over the last 17 years [4]. Thus, it is vital to develop a more accurate disease monitoring model for winter wheat to prevent the occurrence of powdery mildew.

The main periods of the wheat powdery mildew cycle include over-summering, autumn seedling infection, overwintering, and spring epidemics. Over-summering is known to be a key period in all epidemic processes [5]. In Shaanxi Province, China, wheat powdery mildew can survive during summer and winter. The disease completes its yearly infection cycle in the northern and southern mountains and in the Guanzhong plains. The pathogen’s conidial spores and ascospores can infect volunteer wheat during over-summer periods [6]. Moreover, meteorological conditions can affect the prevalence and damage caused by powdery mildew, especially during the over-summering period [5].

Crop diseases often induce physiological changes in plant metabolism, causing variations in plant pigment and water content, as well as changes in cell structure, which can in turn cause changes in crop reflectance [7,8]. For example, increased reflectance in the visible bands is associated with the breakdown of chloroplasts and visible foliar symptoms [9,10]. The increase in reflectance in the mid-infrared and shortwave near-infrared bands indicates water deficiency [11,12]. In crop disease research, remote sensing technologies have mainly been used for disease detection, monitoring, identification, and differentiation, and crop diseases have been successfully identified and differentiated based on hyperspectral reflectance data [4,13,14]. Hyperion satellite hyperspectral imagery has also been evaluated for the potential to detect plant disease [15]. Although the hyperspectral system gives more detailed information for identifying feature bands responding to particular crop diseases, its application over large scales is difficult due to its high hardware and computational costs. Therefore, multispectral sensing systems with 3~6 broad bands (ranging from visible to near-infrared spectral regions) has developed as an alternative technology and has been widely used to explore wheat characteristics and habitat traits [8]. A number of studies have demonstrated the use of satellite imagery for disease monitoring. For instance, SPOT-6, Worldview-2, HJ, and Landsat 8 satellite images were all successfully used for mapping crop diseases, predicting forest pests, etc. [8,16,17,18,19]. Some crop diseases and pests were predicted more successfully by integrating satellite imagery into the meteorological data based prediction models [3,20,21]. These results have encouraged us to use satellite imagery for monitoring the occurrence of powdery mildew in winter wheat. However, for the remote monitoring of crop diseases, most scholars have focused on detection and monitoring of late periods of infection using corresponding single-date imagery. Relatively few studies have considered the use of temporal information. Although some scholars successfully monitored powdery mildew in winter wheat using multi-temporal satellite imagery, the images were only focused on the late disease development period and excluded early growth information [22].

The spatial information for crop disease occurrence and development and their temporal characteristics are crucial for disease monitoring. Some scholars have successfully applied time series images to the detection of tree mortality in forests caused by diseases and pests [23,24,25]. Those results revealed the potential of multi-date image approach in disease monitoring research. The remotely sensed indices of a single-date image which collected at a single time point only reflect the partial characteristics of crop disease because that the powdery mildew occurs throughout the entire wheat growth period from infestation to manifestation [5,6,26,27]. Therefore, we speculated that remote sensing images of the early key infection periods contained useful information on the development of infection, and integrating this information with disease monitoring would effectively improve the performance of the monitoring model.

The *k*-nearest neighbors (KNN) algorithm [28,29,30] is a method for classifying objects based on closest *k* training samples in the feature space. This instance-based learning algorithm is very simple, popular, efficient and effective for pattern recognition, and has been widely used for classification [30,31,32]. The KNN method has also been used in the identification of crop diseases [31,32]. However, there are very few studies that explore whether the KNN classification algorithm combined with the multi-temporal satellite imagery can effectively map crop disease occurrence.

In this study, we used early multi-temporal satellite imagery to develop a model that focuses on monitoring the occurrence of powdery mildew in the late winter wheat growth period (filling period) at regional scales. Multi-temporal Landsat-8 imagery was adopted in this study. The objectives of this study were (1) to identify the optimal multi-temporal data set for the monitoring of powdery mildew occurrence in winter wheat from a number of different multi-temporal combinations; (2) to assess the feasibility of using imagery containing information for early critical disease infection periods to monitor the occurrence of powdery mildew in late winter wheat growth period; (3) to evaluate the performance of the multi-temporal indices-based KNN disease monitoring approach and its capability for mapping powdery mildew occurrence.

## 2. Materials and Methods

### 2.1. Study Sites and Disease Field Survey

**Figure 1:**
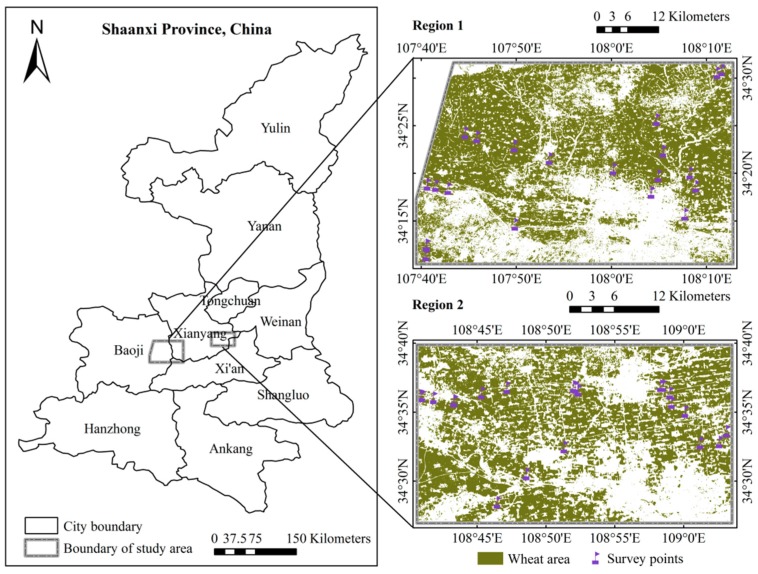
Geographic location and spatial distribution of wheat areas and sample points.

The study area encompassed two typical regions affected by the disease (region 1 and region 2), located in the western Guanzhong plains, Shaanxi Province, China (Figure 1). The area was located in a high-yield farming area with good hydro-thermal conditions, along with a mild and humid climate. In this region, there are four seasons, precipitation is concentrated, and rainfall and temperature are synchronized. The average annual temperature ranges from 9.9 to 15.8 °C. The average annual rainfall ranges from 500 to 700 mm, and rainfall decreases from west to east, and from south to north. The average annual evaporation ranges from 1000 to 1200 mm, and the frost-free period spans from 130 to 220 days [33]. Winter wheat is a major local crop, and the area provides a suitable propagating and developing environment for the powdery mildew pathogen.

A total of 62 field survey plots were collected to evaluate the damage severity caused by winter wheat powdery mildew as ground truth data in region 1 and region 2 during 10 May 2014 (Figure 1). Five 1-m × 1-m ranges were uniformly selected at a 30-m × 30-m spatial extent to match the disease field investigation and the spatial resolution of Landsat-8 satellite imagery. The central latitude and longitude of each plot were recorded by a sub-meter precision handheld Global Positioning System (GPS). Wheat growth conditions, height, and occurrence severity were noted in the survey. According to the National Rules for the Investigation and Forecasting of Crop Diseases (NY/T 613-2002), each leaf of the selected plants were grouped into one of 10 levels: 0 (amount of infection: 0%), 1 (1–10%), 2 (11–20%), 3 (21–30%), 4 (31–40%), 5 (41–50%), 6 (51–60%), 7 (61–70%), 8 (71–80%), 9 (81–100%). Of them, 0% represents no infection and 100% represents the greatest amount of infection. Then the *DI* was calculated using:

(1) $$DI=\frac{\sum xf}{n\sum f}\times 100$$

where *x* is the value of incidence level, *f* is the total number of leaves for each degree of disease severity, and *n* is the value of highest disease severity gradient.

For simplicity, in this study, disease occurrence severity was grouped into three classes for subsequent analysis. These classes included: normal (*DI* = 0), slight infection (0 < *DI* ≤ 30%), and severe infection (*DI* > 30%). The criteria of *DI* = 30% for these classifications was suggested by China’s national plant protection department (NY/T 613-2002) [22]. The overview of the field survey is listed in Table 1. Region 1 is a typical occurrence area of wheat powdery mildew, the investigations in this region contained a complete disease severity. Hence, data collected in this region was used to model and calibrate the optimal multi-temporal combination. Alternatively, region 2 represented the general situations of wheat powdery mildew and is more in line with the actual situation of the field in non-large occurrence years, thus, the levels of *DI* are generally lower than 3. Therefore, data collected in region 2 was just used for validating in this case.

### 2.2. Image Acquisition and Preprocessing

Powdery mildew is wind-dispersed and infects volunteers after harvest and autumn-sown crops. The disease cycles slowly but can continue during the winter if temperatures are mild. In spring, the growth increases rapidly in tandem with rising temperature and high humidity, and infects leaves [26,27]. Generally, a powdery mildew hypha recovers growth during the first ten days in February. The development period of the disease begins in March and occurs in April and May [34]. Considering the infection, occurrence, and development characteristics of powdery mildew, six temporal Landsat-8 images were acquired during November, 2013 to May, 2014. These images were used to combine early growth information for winter wheat and for monitoring powdery mildew in the late critical development period. The development period refers to the occurrence of wheat powdery mildew in May, or the filling period of winter wheat. The details regarding the dates and periods for the images are provided in Table 2. A radiometric calibration and an atmospheric correction for Landsat-8 images were conducted using ENVI 5.3 software.

### 2.3. Planting Area Extraction of Winter Wheat

The spectral divergences among different ground objects, such as farmland, forests, water bodies, and impervious areas are always greater than that between healthy and diseased areas in crop fields. Thus, it is necessary to extract information from the winter wheat planting area before conducting disease monitoring, and a decision tree method was successfully applied to the extraction of winter wheat planting area [22]. Based on the phenological information for the main crops in the study area [35], we used this decision tree method for the classification process. The method was validated using field survey points, and an overall accuracy of 94% for extracting information from the crop area were obtained. The results satisfied the accuracy required for subsequent analysis and disease monitoring.

### 2.4. Remotely Sensed Indices Extraction for Disease Monitoring

It is reported that the plant growth status and habitat characteristics are associated with the susceptibility of plants to diseases, and the physiological and biochemical characteristics will be changed once the plant is infected by the diseases [3,8,36,37]. Therefore, in this study, four remotely sensed indices which related to crop vigor, water status and stress were selected to investigate their sensitivity for winter wheat powdery mildew. These indices included: the triangular vegetation index (TVI), optimized soil adjusted vegetation index (OSAVI), disease water stress index (DSWI), and shortwave infrared water stress index (SIWSI). The TVI is known to be a good estimation index for the leaf area index (LAI), and it is also sensitive to chlorophyll content [38]. The OSAVI was selected to minimize brightness-related soil effects, and it is an optimization of the soil adjusted vegetation index (SAVI) [39,40]. The loss of moisture due to lesions or ruptured leaves is an important factor in disease detection [41]. Thus, the DSWI and SIWSI indices were included to capture the plant’s water status. Both indices contain NIR and SWIR bands and have potential for detecting water stress in crops at the canopy level [3,42]. The formulas for these vegetation indices (VIs) are provided in Table 3. In total, 24 VIs (four VIs of each of the six periods) were extracted using the six temporal Landsat-8 images.

### 2.5. Disease Occurrence Monitoring Using k-Nearest Neighbors (KNN)

The KNN algorithm was implemented to monitor powdery mildew based on multi-temporal Landsat-8 imagery and its performance was evaluated. This algorithm consists of training phase and classification phase. In training phase, the training examples are vectors (each with a class label) in a multidimensional feature space. The feature vectors and class labels of training samples are stored in this phase. In the classification phase, *k* is a user-defined constant, a query or test point (unlabeled vector) is classified by assigning a label, which is the most recurrent among the *k* training samples nearest to that query point. No actual model or learning is performed during the training phase, although a training data set is required, it is used solely to populate a sample of the search space with instances whose class is known, for this reason, this algorithm is also known as lazy learning algorithm. It means that the training data points are not used to do any generalization and all the training data is needed during the testing phase. When an instance whose class is unknown is presented for evaluation, the algorithm computes its *k* closest neighbors, and the class is assigned by voting among those neighbors [45]. The main advantage of the KNN algorithm is that it performs well with multi-modal classes since its decisions are based on a small neighborhood of similar objects. Therefore, the algorithm can still provide good accuracy even if the target class is multi-modal [46]. One of the key factors determining the performance of the KNN algorithm classification is the measure of the distance. Euclidean distance is the most common distance function for the KNN, but it is easily influenced by the mode characteristic dimension [47]. Compare to Euclidean distance, Pearson Correlation is not sensitive to magnitude [48]. Thus, we used Pearson Correlation as the distance function for the KNN algorithm in this study.

### 2.6. Optimal Multi-Temporal Combination for Monitoring Model

Although VIs of the six different periods were chosen as the primary input variables for the construction of multi-temporal VIs-based KNN monitoring model, whether the performance of this multi-temporal combination was optimal or not was uncertain. Hence, a back stepwise elimination [49] method was used to identify the optimal multi-temporal data set for disease monitoring. First, 24 VIs (that is, four VIs of each of the six periods) were included as input variables of the KNN monitoring model. Then, the backward stepwise elimination method started with these VIs based on the KNN model, with the four VIs of one period subsequently being eliminated at a time. The elimination started with 1 and proceeded sequentially. At each step, deleted variables are those that result in low performance of the model. Otherwise, the variables are retained as an improvement to the model, and a new selection cycle begins. This method of deletion continues until only one period’s variables are left in the model or until a stopping rule is satisfied. The coefficient of determination (R^2^) and the root mean square error (RMSE) were used as accuracy measurements of multi-temporal combination group estimation.

### 2.7. Accuracy Assessment of Disease Monitoring

**Figure 2:**
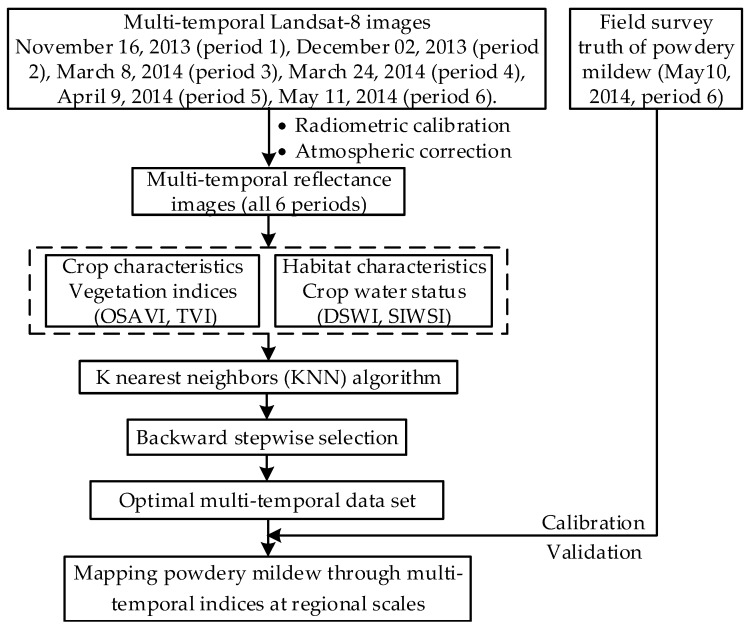
Flowchart for constructing monitoring models for powdery mildew using Landsat-8 imagery at regional scales.

Several statistical accuracy indicators (Somers’ D, Kendall’s Tau-c, Goodman-Kruskal Gamma, and Spearman correlation) were used to evaluate the performance of the KNN disease monitoring models. Additionally, an overall accuracy (OA), user’s accuracy (UA), producer’s accuracy (PA), and kappa coefficient were calculated for the monitoring results based on field truths. These accuracy assessments were used to evaluate the model’s performance in monitoring disease occurrence severity. For comparison and validation, a classification and regression tree (CART) [50] and back propagation neural networks (BPNN) [51] were also used. To further compare and verify the performance of the multi-temporal VIs-based monitoring models, the traditional single-date (period 6) VIs-based monitoring models were also established using CART, BPNN, and KNN methods. The three methods models were calibrated using disease data collected from region 1 and validated by disease data collected from region 2. Figure 2 summarizes the data analysis process. The calibration and validation of the three approaches were conducted using MATLAB 2016a software. The CART monitoring model was calibrated using the SPSS Clementine 12.0 software.

## 3. Results

### 3.1. Response of VIs to Powdery Mildew and Its Development

**Figure 3:**
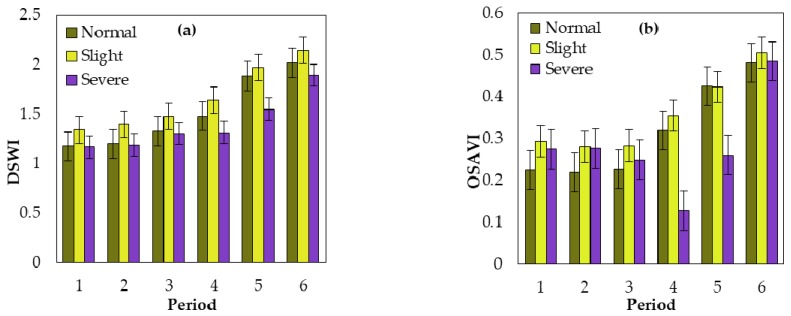

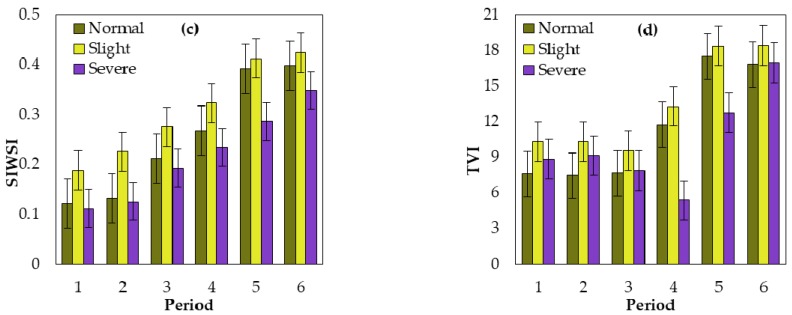
Mean and standard deviations of the (**a**) DSWI, (**b**) OSAVI, (**c**) SIWSI, and (**d**) TVI for both normal and infected (slight and severe) plots at different periods.

The spectral changes induced by powdery mildew infection forms the basis for remote sensing monitoring. Generally, the three visible channels (i.e., blue, green, and red bands) display a higher reflectance in diseased samples than in healthy samples, but the NIR channel exhibits the opposite pattern [22]. This spectral responses’ abnormality was resulted by the changes of biophysical and biochemical parameters of plants induced by the crop disease pathogens, such as variations of several pigments, water content and canopy structure, as well as some leaf color changes due to pustules or lesions [10,52]. In this study, four selected indices all contained a visible band or NIR band. Figure 3 displays the difference in the response indices among normal, slightly, and severely infected plots. The indices were compared at all six growing periods using the mean and standard deviation of each index. All six temporal indices provided the highest values for slightly diseased samples with three disease occurrence severity (i.e., normal, slight, and severe).

The DSWI and SIWSI provided the lowest values for severely diseased samples in six periods. The OSAVI and TVI provided the lowest values for normal samples in the first three periods, and which provide the lowest values for severely diseased samples during the remaining three periods. Additionally, the indices among the three samples exhibited different changing patterns over time.

### 3.2. Optimal Multi-Temporal Data Set for Mapping Powdery Mildew

For backward stepwise elimination analysis, the results evaluation of different multi-temporal combination groups are listed in Table 4. The results illustrate that the multi-temporal VIs-based model composed of six periods had the lowest R^2^ and highest RMSE. Meanwhile, the other period groups’ assessment results showed that removing VIs of period 1 and period 3 significantly improved the performance of the model. Additionally, the remaining four periods (periods 2, 4, 5, and 6) were all important; the absence of any one period VIs would lead to a decline in the accuracy of the model. Therefore, the VIs of periods 2, 4, 5, and 6 composed the optimal multi-temporal input variables for disease monitoring model.

### 3.3. Mapping Powdery Mildew through Multi-Temporal Indices

**Figure 4:**
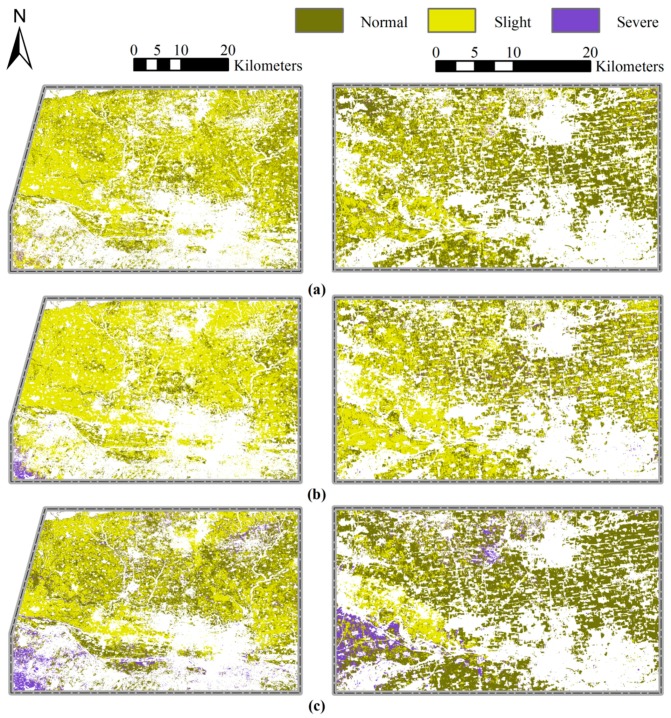
Maps of powdery mildew occurrence severity in winter wheat produced by the (**a**) CART, (**b**) BPNN, and (**c**) KNN models using optimal multi-temporal VIs.

Optimal multi-temporal (including periods 2, 4, 5, and 6) VIs-based models were constructed using the CART, BPNN, and KNN methods to detect the importance of early period information for disease monitoring. In the three multi-temporal models, powdery mildew occurred extensively in region 1, and slightly occurred in region 2 (Figure 4). The monitoring results were supported by field observations. In region 1, 72% of the plots (44% slightly infected and 28% severely infected) were infected with powdery mildew. Less than 20% of the plots were slightly infected with the disease in region 2. The disease infection maps revealed that the area in region 1 had a higher occurrence severity. In region 2, the results indicated a higher disease infection severity in the western area (Figure 4). The results from the three models were compared, and the KNN model revealed the highest amount of severe infection. Moreover, the BPNN model revealed the highest amount of slight disease infection. A quantitative evaluation of the three models using multi-temporal VIs revealed that all three models produced high statistical parameters. The KNN model featured the highest Somers’ D, Kendall’s Tau-c, Goodman-Kruskal Gamma, and Spearman correlation (Table 5). The performance of the multi-temporal VIs-based models were assessed using field survey truthing in region 2 (Table 6). Generally, the powdery mildew-infected areas were successfully monitored by all three methods using multi-temporal VIs, although the KNN models had obtained the highest OA of 84.6% and the highest kappa coefficient of 0.516 among three approaches.

**Figure 5:**
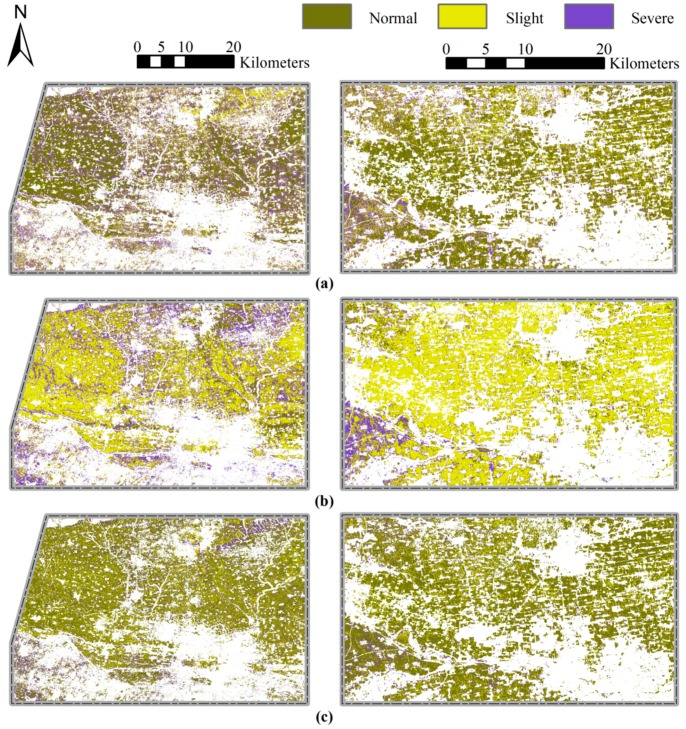
Maps of powdery mildew infection in winter wheat produced by the (**a**) CART, (**b**) BPNN, and (**c**) KNN models using traditional single-date VIs.

The CART, BPNN, and KNN monitoring models were also constructed through traditional single-date VIs (period 6), and used to map powdery mildew for region 1 and region 2 (Figure 5). These maps were assessed and a significant difference were observed among the different methods (Figure 5).

For example, normal and severely infected wheat were distributed throughout the study area in the CART single-date VIs-based model. In the BPNN and KNN models, slightly infected and normal wheat covered most of the entire crop area. Several statistical parameters were used to evaluate the performance of the single-date VIs-based models (Table 7). The CART and BPNN models displayed statistical parameters with low values. Further, validation results using field survey truthing revealed that only the KNN model had an acceptable monitoring OA of 76.9% (Table 8).

Compared to the traditional single-date VIs, all three models produced higher statistical parameters for multi-temporal VIs. The multi-temporal VIs models produced the higher values for Somers’ D, Kendall’s Tau-c, Goodman-Kruskal Gamma, and Spearman correlation. However, the BPNN method had lower values for Kendall’s Tau-c and Goodman-Kruskal Gamma. The results can be seen in Table 6 and Table 8. In the three multi-temporal VIs-based models, the KNN model produced the highest statistical parameters, followed by the BPNN and CART models (Table 6). The validation results revealed that the powdery mildew infected areas were successfully monitored by all three methods using multi-temporal VIs. In the single-date VIs-based models, only the KNN method provided an acceptable overall monitoring accuracy (Table 6 and Table 8). The accuracy indicators varied significantly for the three methods when using different data sources. For example, the incorporation of early period winter wheat growth information increased the overall accuracy of the CART, BPNN, and KNN models from 34.6% to 73.1%, 50.0% to 80.8%, and 76.9% to 84.6%, respectively. Furthermore, the kappa coefficients for the BPNN, and KNN models improved from 0.201 to 0.330, and 0.355 to 0.516. The KNN model outperformed both the CART and BPNN models using both single-date and multi-temporal VIs.

## 4. Discussion

We found that classification using multi-temporal VIs produced higher accuracies than the traditional single-date VIs when monitoring for powdery mildew in winter wheat. Not all selected periods were useful for monitoring of disease; in six different periods, only the VIs of period group which composed with periods 2, 4, 5, and 6 were the optimal multi-temporal data set for monitoring of powdery mildew in winter wheat. Furthermore, only the KNN model obtained acceptable results among the three methods using traditional single-date VIs. The KNN method was also observed to produce the most accurate classification using the multi-temporal VIs approach. The overall monitoring accuracy of multi-temporal VIs was obviously higher than that of the traditional single-date VIs. This suggests that the methodology employed by the multi-temporal VIs improved overall monitoring accuracy.

Winter wheat undergoes a series of physiological and biochemical changes when infected with powdery mildew. These changes can alter the spectral response characteristics of winter wheat in the visible and NIR spectral ranges [8]. For instance, compared to normal wheat leaves, a significant increasing of the raw reflectances of diseased leaves has been found in the visible spectral region, while a slight decrease was appeared in the NIR region. Meanwhile, for diseased leaves, the “blue shifiting” phenomenon of red edge positions is significant [53]. In this study, the chosen remotely sensed VIs (i.e., DSWI, OSAVI, SIWSI, and TVI) exhibit remarkable performance on monitoring of powdery mildew occurrence. These VIs enable transformation of raw spectra into more meaningful metrics of disease severity. Existing research indicates that TVI and OSAVI are both suitable for representing crop characteristics [38,39,42] while DSWI and SIWSI are both sensitive to water stress in crops at the canopy level [42], which explained their good performance in the monitoring of powdery mildew occurrence.

Six periods for the extraction of VIs in this study represent the development of powdery mildew infection in winter wheat [26,34]. The use of backward stepwise elimination method demonstrated that period 1 and period 3 had negative response in multi-temporal VIs-based models, and the optimal multi-temporal period group was the composition of periods 2, 4, 5, and 6. The possible reason for the negative contribution of period 1 is that the wheat plants in period 1 were small, the VIs affected seriously by the soil background due to the low vegetation coverage [54,55]. On the other hand, the result also indicated that period 1 may not be sensitive to wheat powdery mildew. Both period 3 and 4 were in the wheat re-greening period (Table 2), but the performance of the two periods in the multi-temporal monitoring model was completely different (Table 4), which indicated that period 4 was more sensitive to wheat powdery mildew than period 3. It is speculated that the winter wheat phenological characteristics were the cause of this phenomenon. In the early March (period 3), winter wheat just entered the re-greening period from the wintering period, the wheat vigor just began to recover and its nutritional status was poor, which inhibited the development of the pathogens. By the late March (period 4), winter wheat had grown vigorously, and the pathogens also became active. This inference is consistent with the infection cycle of wheat powdery mildew [26,27,34]. In our study area, no matter from our result or local winter wheat biological characteristics, both period 1 and period 3 had little contribution to the occurrence and development of the disease. However, our method is just a case analysis, the key periods of different areas would be different due to the effect of local crop phenological characteristics. Therefore, the robustness of our approach needs to be further validated in future.

In this study, KNN method has been successfully used for the monitoring of powdery mildew occurrence in winter wheat. No matter based on single-date or multi-temporal VIs, the KNN method outperformed the CART and BPNN methods, which demonstrated that the KNN [56] model performed better in describing the local characteristics of wheat field than other two methods (Figure 4 and Figure 5). For the disease monitoring, the positive contribution of the early wheat growth information was confirmed in this research by developing models using single-date and multi-temporal VIs. For the three methods, compared with the single-date based models, the overall accuracies of the homologous multi-temporal based monitoring models increased by 38.5% (CART), 30.8% (BPNN), and 7.7% (KNN), respectively. It is reported that there were other factors leading to responses of the same spectral indices apart from the disease infestations [57]. Hence, the result demonstrated that the multi-temporal VIs not only enhanced better the wheat disease information, but also eliminated effectively the fluctuation of indices caused by the phenology, cultivation, and plant condition differences between fields within the single-date scene. Moreover, compared with the traditional single-date VIs, the multi-temporal VIs set produced more reliable nearest neighbor decision for KNN classification. Undesirably, all three methods had a poor PA (maximum value = 60.0%) for the validation results for slight disease infection in region 2. This suggests that the methods were unable to monitor lower infection severity. Different winter wheat cultivars have different response to powdery mildew infection [58], and intercropping can also influence the occurrence of diseases [59]. However, the field management (i.e., cultural, phytosanitary treatments, etc.) and wheat variety information across our study area was very complicated, and the resistance or susceptibility between different varieties was only roughly reflected in the disease severity in our study. Therefore, in future, more detailed research through control experiments which include information on pathogen spore concentration, the susceptibility of winter wheat cultivars, and field management should be considered to improve the model accuracy and reliability.

Overall, the multi-temporal VIs-based KNN model performed better than existing traditional models in monitoring occurrence of powdery mildew in winter wheat based on Landsat-8 imagery data, with a good accuracy of 84.6%. We expect the selection periods to include the whole key periods of powdery mildew development and to observe the disease’s entire evolution in the entire growth period of winter wheat. However, due to data limitations, the characteristics of remote sensing in critical periods of disease infestation were only considered from a phenological perspective in this study. The occurrence, development, and dispersal of plant diseases are closely associated with local weather conditions [60,61,62]. The disease develops rapidly once the weather satisfies certain conditions and it is inhibited if the weather conditions are not suitable. On the other hand, ground surveys only collected the powdery mildew occurrence severity in filling period (on May 10 2014, period 6), there were no corresponding disease occurrence and development situations of other wheat growth periods. Therefore, our model only monitored the late development of wheat powdery mildew, but the entire evolution of the disease was not observed. In future research, more disease fieldwork of different wheat growth periods should be carried out, and the relationship between early growth information of wheat (i.e., habitat, meteorological conditions, etc.) and disease infestation and evolution should also be analyzed.

## 5. Conclusions

This study developed a multi-temporal VIs-based KNN approach based on Landsat-8 imagery to monitoring powdery mildew in winter wheat. The performance of this approach was evaluated from two aspects, for one thing, by compared with traditional single-date based methods, the accuracy of the proposed approach had increased by 7.7%, for another, we also compared this method with the CART, BPNN, and KNN, which suggested that the multi-temporal VIs-based KNN method has an excellent performance in monitoring powdery mildew in winter wheat at the regional scale, with the overall accuracy of 84.6%. This approach provides an evidence for using the satellite observations on guiding field disease prevention and management. Our future work would focus on: (1) the detection and classification of multiple diseases at the field scale, and (2) the relationship analysis between early growth information of wheat (i.e., habitat, meteorological conditions, etc.) and disease evolution.

## Figures and Tables

**Table 1:** Basic information for the disease survey experiment.

Location	Type	Number of Field Survey Samples			
		Normal	Slight	Severe	Sum
Region 1	Calibration	10	16	10	36
Region 2	Validation	21	5	0	26

**Table 2:** Information provided by the images for disease monitoring.

Growth Period	Period Number	Image Acquisition Date
Wintering period	Period 1	16 November 2013
	Period 2	2 December 2013
Re-greening period	Period 3	8 March 2014
	Period 4	24 March 2014
Jointing period	Period 5	9 April 2014
Filling period	Period 6	11 May 2014

**Table 3:** Summary of the spectral vegetation indices used for monitoring of powdery mildew, with red band, green band, NIR band, and SWIR band denoted as R_R_, R_G_, R_NIR_, and R_SWIR_, respectively.

Title	Definition	Formula	Reference
DSWI	Disease water stress index	(R_NIR_ + R_G_)/(R_SWIR_ + R_R_)	[43]
OSAVI	Optimized soil adjusted vegetation index	(R_NIR_ − R_R_)/(R_NIR_ + R_R_ + 0.16)	[40]
SIWSI	Shortwave infrared water stress index	(R_NIR_ − R_SWIR_)/(R_NIR_ + R_SWIR_)	[44]
TVI	Triangular vegetation index	0.5 × (120 × (R_NIR_ − R_G_) − 200 × (R_R_ − R_G_))	[38]

**Table 4:** Estimating results for monitoring models based on different multi-temporal combination groups.

Period Group	Periods 1 to 6	Periods 2 to 6	Periods 3 to 6	Periods 2, 4, 5, 6	Periods 2, 5, 6	Periods 2, 4, 6
R^2^	0.50	0.69	0.61	0.79	0.53	0.72
RMSE	0.58	0.44	0.51	0.36	0.55	0.42

**Table 5:** Statistical measures of the goodness of fit for the optimal multi-temporal VIs-based CART, BPNN and KNN monitoring models.

Method	Statistical Parameters			
	Somers’ D	Kendall’s Tau-c	Goodma-Kruskal Gamma	Spearman Correlation
CART	0.305	0.225	0.655	0.309
BPNN	0.332	0.189	0.727	0.333
KNN	0.505	0.314	0.869	0.505

**Table 6:** Validation of the optimal multi-temporal VIs-based models using field truth samples in region 2.

Validation		Field Truth					
Method		Normal	Slight	Sum	UA	OA	Kappa
CART	Normal	16	2	18	88.9%	73.1%	0.295
	Slight	5	3	8	37.5%		
	Sum	21	5	26			
	PA	76.2%	60.0%				
BPNN	Normal	19	3	22	86.4%	80.8%	0.330
	Slight	2	2	4	50.0%		
	Sum	21	5	26			
	PA	90.5%	40.0%				
KNN	Normal	19	2	21	90.5%	84.6%	0.516
	Slight	1	3	4	75.0%		
	Severe	1	0	1			
	Sum	21	5	26			
	PA	90.5%	60.0%				

**Table 7:** Statistical measures of the goodness of fit for the traditional single-date VIs-based CART, BPNN, and KNN models.

Method	Statistical Parameters			
	Somers’ D	Kendall’s Tau-c	Goodma-Kruskal Gamma	Spearman Correlation
CART	0.109	0.107	0.257	0.124
BPNN	0.263	0.207	0.778	0.274
KNN	0.361	0.254	0.729	0.364

**Table 8:** Validation of the traditional single-date VIs-based models using field truth samples in region 2.

Validation		Field Truth					
Method		Normal	Slight	Sum	UA	OA	Kappa
CART	Normal	7	1	8	87.5%	34.6%	0.035
	Slight	8	2	10	20.0%		
	Severe	6	2	8			
	Sum	21	5	26			
	PA	33.3%	40.0%				
BPNN	Normal	8	0	8	100%	50.0%	0.201
	Slight	12	5	17	29.4%		
	Severe	1	0	1			
	Sum	21	5	26			
	PA	38.1%	100%				
KNN	Normal	17	2	19	89.5%	76.9%	0.355
	Slight	4	3	7	42.9%		
	Sum	21	5	26			
	PA	81.0%	60.0%				
